# Supplementary figures and images for: CRISPR/Cas9 mutagenesis against sex pheromone biosynthesis leads to loss of female attractiveness in Spodoptera exigua, an insect pestt
Source: PLoS One. 2021 Nov 17;16(11):e0259322. doi: 10.1371/journal.pone.0259322 (PMC8598075; doi:10.1371/journal.pone.0259322)

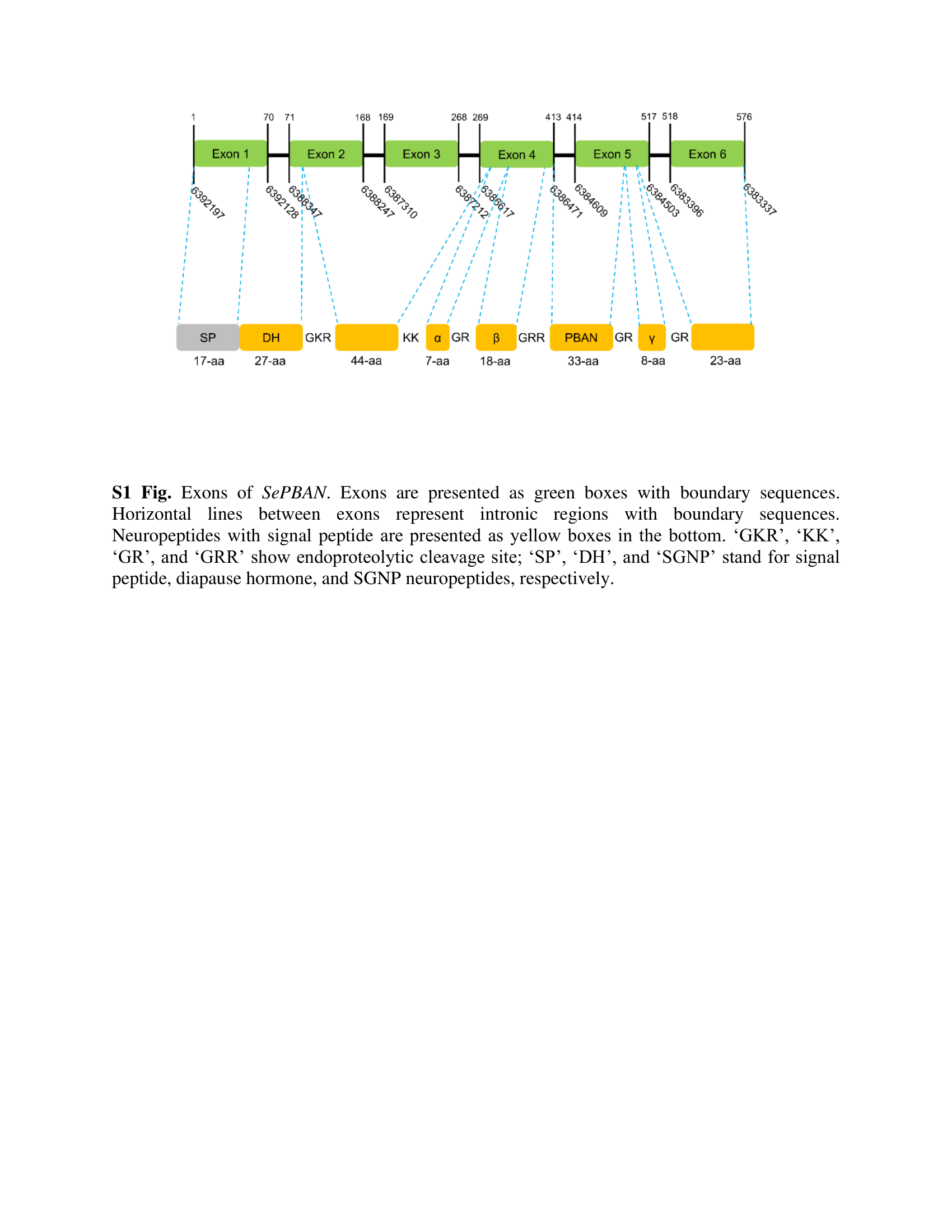

Supplement: S1 Fig — Exons are presented as green boxes with boundary sequences. Horizontal lines between exons represent intronic regions with boundary sequences. Neuropeptides with signal peptide are presented as yellow boxes in the bottom. ‘GKR’, ‘KK’, ‘GR’, and ‘GRR’ show endoproteolytic cleavage site; ‘SP’, ‘DH’, and ‘SGNP’ stand for signal peptide, diapause hormone, and SGNP neuropeptides, respectively. (TIFF) [file pone.0259322.s001.tiff]

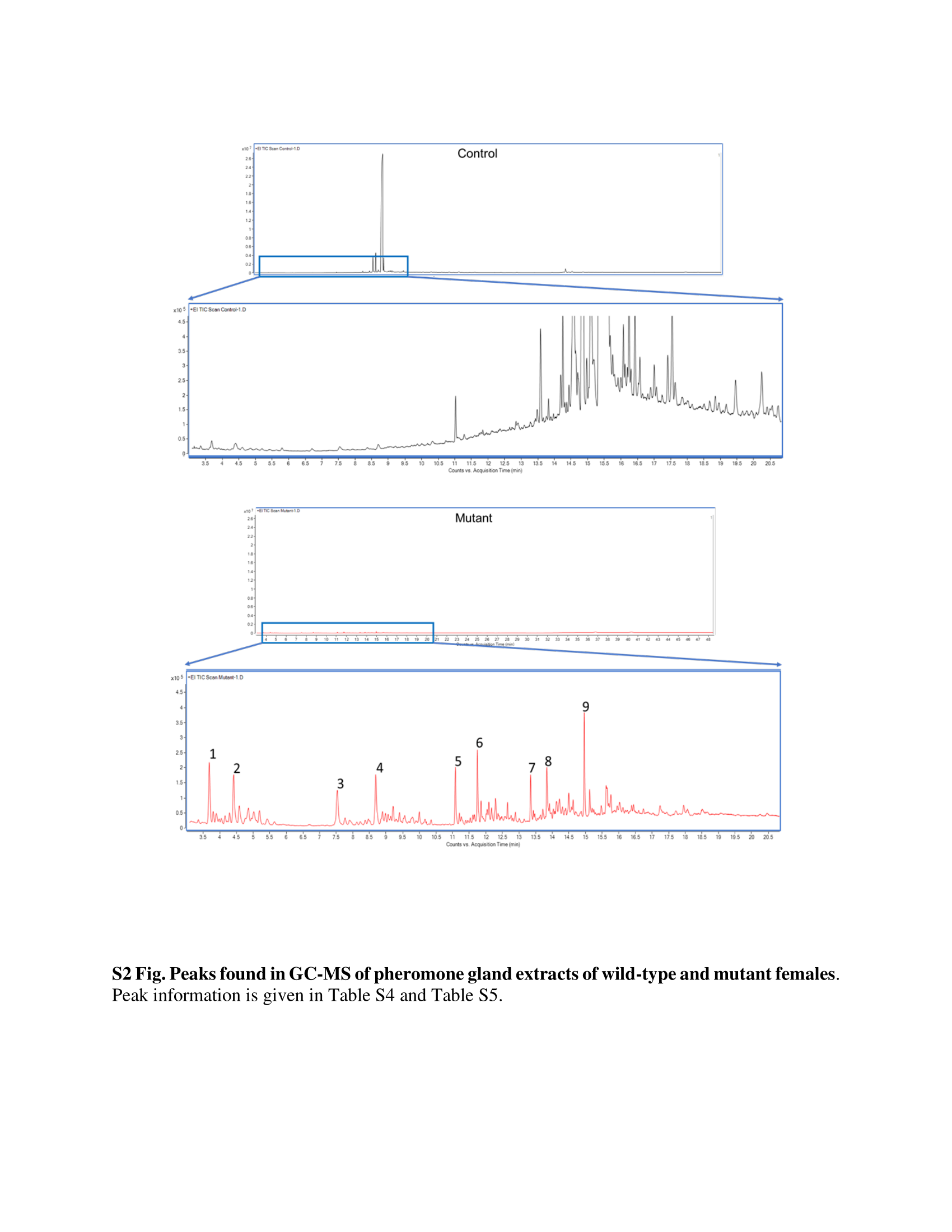

Supplement: S2 Fig — Peak information is given in S3 and S5 Tables. (TIFF) [file pone.0259322.s002.tiff]

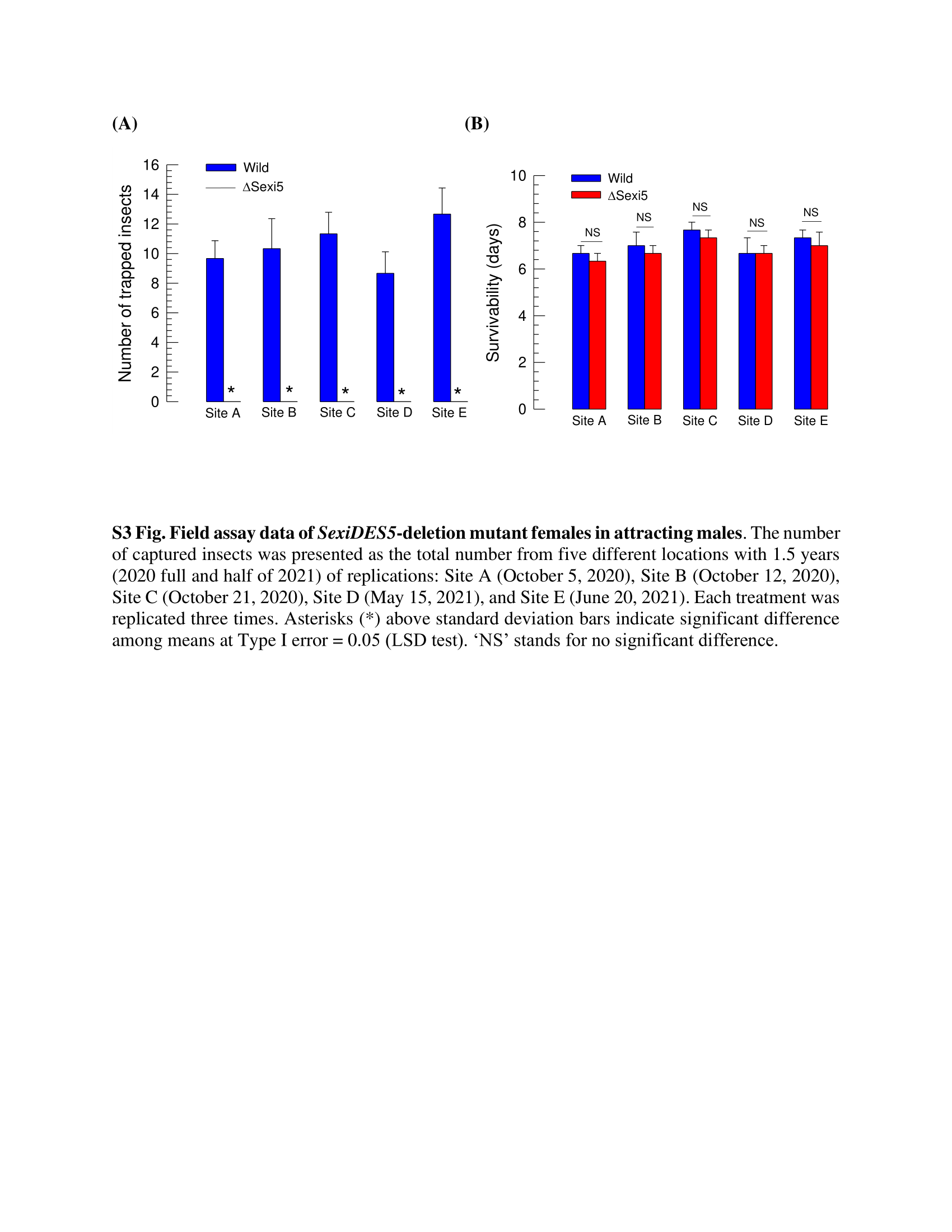

Supplement: S3 Fig — The number of captured insects was presented as the total number from five different locations with 1.5 years (2020 full and half of 2021) of replications: Site A (October 5, 2020), Site B (October 12, 2020), Site C (October 21, 2020), Site D (May 15, 2021), and Site E (June 20, 2021). Each treatment was replicated three times. Asterisks (*) above standard deviation bars indicate significant difference among means at Type I error = 0.05 (LSD test). ‘NS’ stands for no significant difference. (TIFF) [file pone.0259322.s003.tiff]

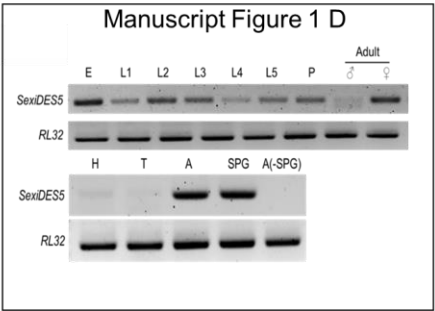

Original figure

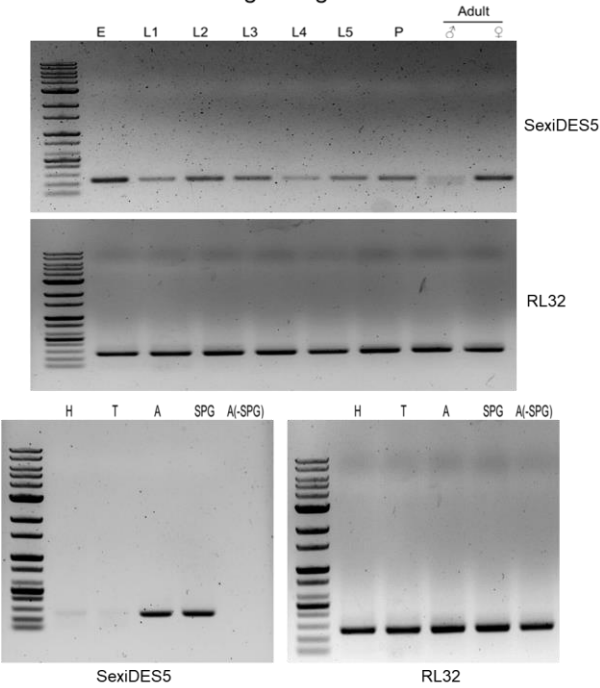

Original figure

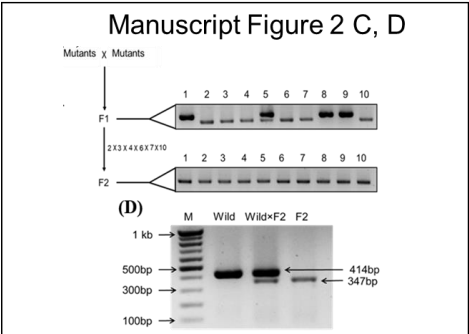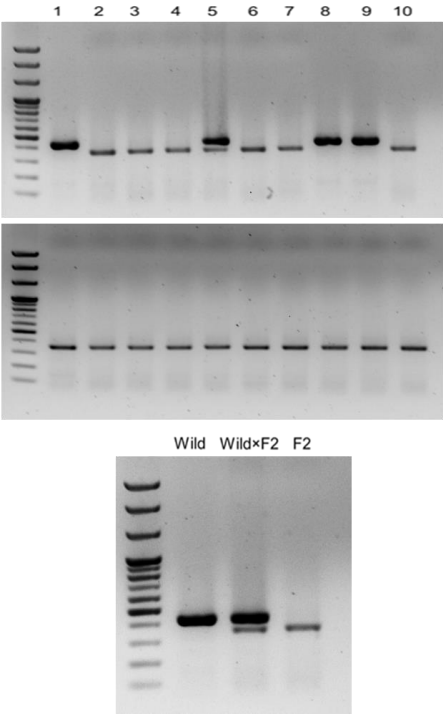

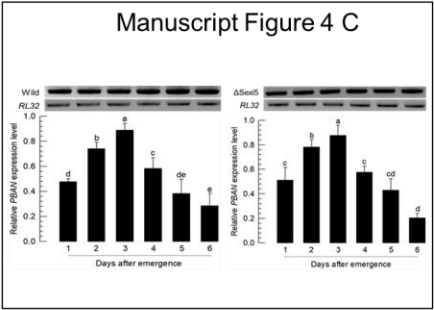

Original figure

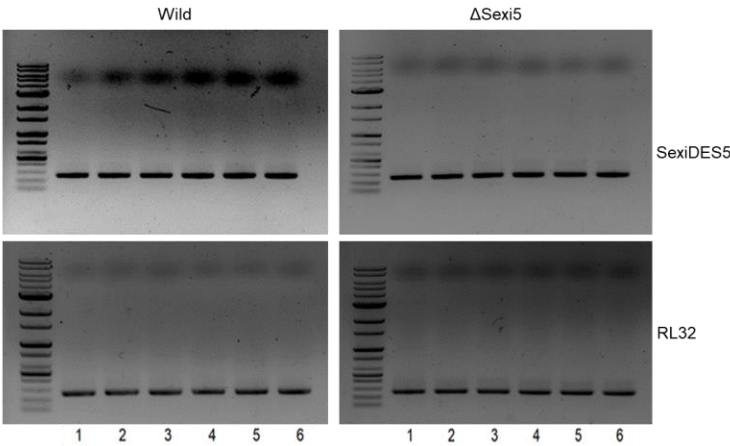

Original figure

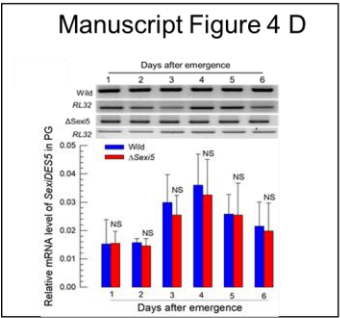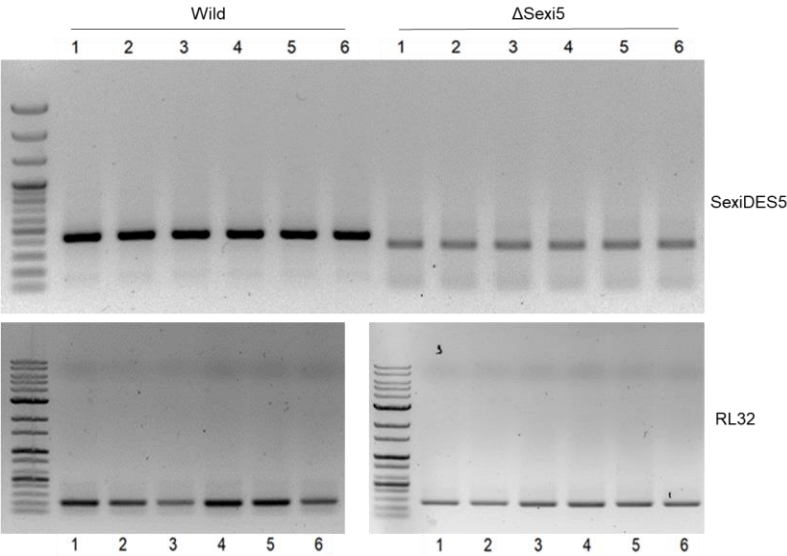

Original figure

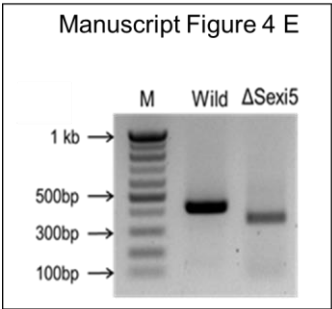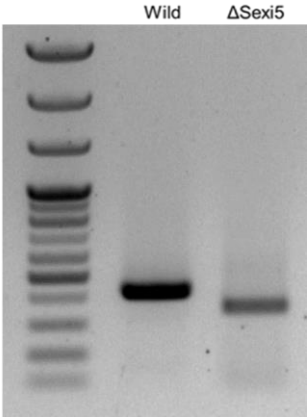

Supplement: S1 File — (PDF) [file pone.0259322.s010.pdf]
