# Supplementary material for: CRISPR/Cas9 mutagenesis against sex pheromone biosynthesis leads to loss of female attractiveness in Spodoptera exigua, an insect pestt
Source: PLoS One. 2021 Nov 17;16(11):e0259322. doi: 10.1371/journal.pone.0259322 (PMC8598075; doi:10.1371/journal.pone.0259322)
Supplement: Attachment — Submitted filename: Response to Reviewers.docx [file pone.0259322.s011.docx]

**Response to reviewers’ comments**

**[Reviewer #1]**

This study investigated the function of SexiDES5 in sex pheromone biosynthesis in Spodoptera exigua. This paper is well written and the data support the conclusions. It is qualified for publishing in PLoS ONE.

**Response**: We appreciate careful review and deeply thank you for your kind encouragement.

**[Reviewer #2]**

**Comment #2-1**: I suggest authors to better explain the reason why SexiDES5 which expressed in all the rest of tested tissues in different developmental stage.

**Response**: We analyzed the gene expression in the whole body of larvae or pupa or adults as described this in the method section and in the figure caption (Fig. 1D). The mutant larvae and pupae showed little aberration in developmental phenotypes compared to wild type except pupal, adult developments and fecundity. The decreased body size and fecundity suggests a role of SexiDES5 other than sex pheromone biosynthesis. The dual role of SexiDES5 catalyzing desaturation suggests its catalysis to produce a PUFA, linoleic acid, which is an essential fatty acid for survival, though it is supplied from diet. We add this explanation based on speculation in the discussion as follows: “Deletion of SexiDES5 did not influence immature developmental rate, although it showed slightly adverse effects on pupal body size and female fecundity. This might be explained by the expression of SexiDES5 in the immature stage through association with de novo synthesis of LA and subsequent polyunsaturated fatty acid (PUFA). SexiDEX5 exhibiting Δ12 desaturation might catalyze the conversion of oleic acid (C18:1) to LA (C18:2). Although LA is usually supplied from diet, its additional de novo biosynthesis would enhance *S. exigua* development and reproduction. LA is known to be a precursor of arachidonic acid, which is the precursor of eicosanoid biosynthesis [30]. Eicosanoids and other PUFAs can mediate various physiological processes including pupal development and adult reproduction [31]. This hypothesis of de novo LA biosynthesis needs to be explored in *S. exigua* in the future.”

**Comment #2-2**: Details of RT-qPCR should be added in Materials and Methods.

**Response**: We previously made a mistake of not including another primer set of *SexiDES5* which was used only to check gene presence by RT-PCR. We included the primer information in S5 Table and details of RT-qPCR is added in the Materials and Methods section as follows: “For checking the presence of *SexiDES5* in the developmental stages and in the different body parts of adults, primer set 1 was used (S5 Table). RT-qPCRs were performed with a qPCR machine (CFX Connect Real-Time PCR Detection System, Bio-Rad, Hercules, CA, USA) using SYBR Green Realtime PCR Master Mix (Toyobo, Osaka, Japan) with gene-specific primers of *SePBAN* or *SexiDES5* (S5 Table) according to the general guideline suggested by Bustin et al. [37]. A ribosomal protein, RL32, gene was used as a control for qPCR because a normalization using this control gene expression demonstrated relative expression of target genes in *S. exigua* [38]. Each cycle was scanned by measuring fluorescence intensity to quantify the PCR products. After the PCR reactions, melting curve analyses were performed from 60^o^C to 95^o^C to ensure consistency and specificity of the amplified products. Each treatment was replicated three times using independent RNA collections. Quantitative analysis of gene expression was done using the comparative CT (2^-ΔΔCT^) method [39]. For checking the effect of CRISPR/Cas9 mediated knocking out of *SexiDES5* on truncated *SexiDES5* mRNA expression, primer set 2 was used (S1 Table).”

**Comment #2-3**: In Table S1, accession number AAT64424 should be corrected AY628764. RL32 gene was used as an internal reference, how can authors be sure that the RL32 is stable in all the samples analysed? Please add reference and the accession number in the text.

**Response**: Accession number is corrected accordingly. Reference is added in line number 339 and the accession number is added in the supplementary table S1.The use of reference gene is explained by the following explanation: “A ribosomal protein, RL32, gene was used as a control for qPCR because a normalization using this control gene expression demonstrated relative expression of target genes in *S. exigua* [38].”

**Comment #2-4**: The manuscript is generally well written with the exception of a few spelling/grammatical errors, the English writing should be further improved.

**Response**: Although this manuscript was cleaned by English-editing company (Harrisco, [www.harrisco.co.kr](http://www.harrisco.co.kr)), the entire text after revision is read by the corresponding author and revised at appropriate.
